# The association of adolescent e-cigarette harm perception to advertising exposure and marketing type

**DOI:** 10.1186/s13690-022-00867-6

**Published:** 2022-04-08

**Authors:** Man Hung, Andrew Spencer, Clarissa Goh, Eric S. Hon, Val Joseph Cheever, Frank W. Licari, Ryan Moffat, Ben Raymond, Martin S. Lipsky

**Affiliations:** 1 College of Dental Medicine, Roseman University of Health Sciences, South Jordan, Utah USA; 2 School of Medicine, University of Utah, Salt Lake City, Utah USA; 3 Department of Psychology, Emory University, Atlanta, Georgia USA; 4 Riverton High School, Riverton, Utah USA; 5 The College, University of Chicago, Chicago, IL USA; 6 Institute of Aging, Portland State University, Portland, Oregon USA

**Keywords:** Marketing, e-cigarette, Harm perceptions, Newspaper, Social media, Health, Adolescents

## Abstract

**Background:**

Despite controversy over their possible health consequences, manufacturers of e-cigarettes employ a variety of marketing media to increase their popularity among adolescents. This study analyzed the relationship between adolescent e-cigarette harm perception and five types of e-cigarette advertising exposures: social media, radio, billboard, newspaper, and television.

**Methods:**

This study used data from Wave 4.5 of the Population Assessment of Tobacco and Health Study (PATH). PATH collects demographic data and interview individuals about issues pertaining to tobacco use, health outcomes, attitudes, and behaviors. This study applied factor analysis to three individual PATH harm perception items to develop a composite harm perception score. Using linear regression, the study explored the relationship of harm perception and participant responses to their recalled viewing of five different types (i.e., newspaper, radio, billboard, television and social media) of advertisements within the past 30 days. A second analysis explored if adjusting for exposure to anti-tobacco messaging and environmental factors such as family approval mitigated the association of harm perception and advertisement types.

**Results:**

The study sample consisted of 12,570 (weighted N = 23,993,149) individuals aged 12 to 17 years old. Unadjusted past 30-day exposure to newspaper, radio, billboard, and social media advertising all correlated with a reduced harm perception, but only the associations for newspaper and social media were statistically significant (*p*<0.05). After adjusting for environmental support factors, exposure to warning labels, and anti-tobacco advertisements, the analysis yielded statistically significant associations between increased e-cigarette harm perception and exposure to radio, billboard, and television advertisements (*p*<0.05). Adjusting for covariates also reduced the association of marketing and harm perception for all forms of media.

**Conclusion:**

E-cigarette advertising influences adolescent perceptions of harm in e-cigarette use, particularly for social media and newspaper advertisements. This association weakens when adjusted for covariates such as environmental support and exposure to anti-tobacco marketing. These findings provide evidence for policy makers to continue anti-tobacco marketing and incorporate environmentally supportive strategies such as holistic, family-centered educational approaches to reduce e-cigarette use among adolescents.

## Background

Over the past decade, adolescent electronic cigarette (e-cigarette) use has increased dramatically. In early 2019, an estimated 27.5% of high school students and 10.5% of middle school students reported e-cigarette use [1]. Many experts argue that marketing tactics used by e-cigarette manufacturers accounts for this increase in popularity. Between 2011 and 2013, adolescent e-cigarette advertisement exposure increased by 256% [2] and almost 70% of middle and high school students reported seeing e-cigarette advertisements in television, local stores or social media [3]. Increased exposure to e-cigarette advertising is linked to increased susceptibility to future product use among never before users, and decreased e-cigarette harm perceptions among both current and never before adolescent users [4–8].

Television, newspapers, and billboards link to reduced e-cigarette harm perception [9]. Social media which enables e-cigarette companies to avoid the increasing marketing restrictions they face with other mediums, is also associated with reduced harm perception and an increased willingness to use e-cigarettes in the future [10, 11]. There appears to be an inverse relationship between all types of marketing and harm perception, with harm perception decreasing as marketing exposure increases [3, 5, 12, 13]. While research indicates that each type of advertisement is independently associated with reduced adolescent harm perception, research comparing the different advertisement types and their influence on harm perception is limited.

In contrast, anti-tobacco marketing can have the opposite effect. Studies found that exposure to anti-tobacco advertisements [14] and health warnings [15] led to increased harm perception and a lower intention to use. Environmental factors can influence behavior as well, with research finding that friend and family approval of e-cigarette use is associated with reduced adolescent harm perception [16]. While both pro and anti-e-cigarette marketing tactics influence adolescent harm perception and susceptibility, no study explores the association between different e-cigarette advertisement types and harm perception while adjusting for exposure to anti-tobacco marketing and environmental support.

This study explored the associations between adolescent e-cigarette harm perception and five separate types of e-cigarette advertising exposure: social media, radio, billboard, newspaper, and television. Additionally, the study examined these associations while controlling for several measures of anti-tobacco exposure and environmental support. Understanding these relationships should assist policy makers in developing strategies to regulate how these products are marketed and to help control adolescent use.

## Methods

### Data source

This study used data from the Population Assessment of Tobacco and Health (PATH) Study, a publicly available database, which is collaboratively sponsored by the National Institute of Health, Center for Tobacco Products, Food and Drug Administration, and the National Institute on Drug Abuse. PATH data consist of both interview and survey questions answered by parents and adolescents. These questions pertain to tobacco use, health outcomes, attitudes, and behaviors and were collected in five waves from 2011 to 2019. PATH sampling uses weighting procedures to adjust for oversampling and nonresponses based on US Census Bureau data to develop nationally representative statistical estimates. The present study utilized a cross-sectional approach using Wave 4.5 data which is the most current available public PATH dataset on youth. More details regarding PATH can be found at https://www.drugabuse.gov/research/nida-research-programs-activities/population-assessment-tobacco-health-path-study.

### Measures

#### Demographics

Sociodemographic variables included parent marital status, household income, youth age at time of interview, race, ethnicity, grade level, and sex.

#### Type of E-cigarette advertisement viewed (predictor variables)

Measures for the type of e-cigarette advertisement viewed came from survey questions in Wave 4.5. Participants were asked whether they recalled viewing five different types (i.e., newspaper, radio, billboard, television and social media) of advertisements within the past 30 days. Participants responded with either “Yes” or “No” for each of the five types.

#### E-cigarette harm perception (outcome variable)

Wave 4.5 participants responded to the following three statements regarding e-cigarette harm perception: (1) Harmfulness of electronic nicotine products to health (Not at all, Slightly, Somewhat, Very, Extremely), (2) Thoughts on how much people harm themselves when they use e-cigarettes or other electronic nicotine products (No harm, Little harm, Some harm, A lot of harm), and (3) Harmfulness of using e-cigarettes or other electronic nicotine products compared to smoking cigarettes (Less harmful, about the same, More harmful).

#### Covariates

The analysis adjusted for exposure and environment factors collected by the PATH survey: the number of friends who use e-cigarettes, views of people close to you about e-cigarettes, how often you’ve noticed health warnings on e-cigarette packs, and how often you’ve seen anti-tobacco marketing in the past 12 months.

### Statistical techniques

Data analyses were completed using SPSS version 28 for Windows. Descriptive statistical measures outlined sociodemographic attributes, outcomes and covariates. Additionally, frequencies for both the unweighted and weighted sample sizes were reported.

Using Kaiser normalization with varimax rotation and principal axis factoring, exploratory factor analysis was performed on the three harm perception variables to assess their factor structure. Factor structure determines whether items are associated with each other to shape an undiscovered model (e.g., factor). If there are related response patterns between each harm perception item, a cumulative measure can be created to assess harm perception more concisely.

Factor structure is determined by factor loading and a single construct is subsequently formed by weighing the correlation between each item. Factor loading values range from -1 to 1, with those larger than |0.4| considered adequate for construct formation [17]. The Kaiser-Meyer-Olkin Measure of Sampling Adequacy tests for the sampling adequacy of selected items and the complete dataset. Within this framework, the study used a value of >0.6 as sufficient for factor analysis and a Barlett’s test of sphericity with *p<0.05* to indicate a correlation between items.

The reliability of the harm perception factor was evaluated using Cronbach’s alpha with a sufficient Cronbach’s alpha value set > 0.6 [18]. Once each factor displayed sufficient reliability and factor loadings, a factor score for the harm perception construct was computed using the weighted average of the items’ scores based on factor loadings. Higher factor scores indicate that adolescents view e-cigarettes as more harmful.

Subsequently, linear regression analysis was applied to test the following research question: Does the type of e-cigarette advertisement viewed associate with adolescent e-cigarette harm perception, with and without adjustment for exposure and environmental factors? A standardized regression coefficient with a 95% confidence interval (CI) was calculated. A two-tailed *p*-value less than 0.05 was considered statistically significant.

## Results

The study sample consisted of 12,570 (weighted N = 23,993,149) individuals aged 12 to 17 years old. Approximately 46.6% were aged 12 to 14 with the remaining 53.4% aged 15 to 17. Among the respondents, 48.1% identified themselves as female, 69.0% as White and 30.4% as Hispanic. The sample group was fairly evenly distributed across grade levels. In the sample group, 63.7% of participants reported having a married parent/guardian, and 29.0% reported an annual household income of >$100,000. Table 1 summarizes the sample group’s characteristics. Among the five advertisement types, radio was the least popular, with only 7.8% reporting past 30-day exposure (n = 980, weighted N = 1,783,605). Conversely, social media was the most popular, with 21.3% of the sample reporting past 30-day exposure (n = 2,688, weighted N = 5,051,394). The other three types - billboard, newspaper and television - fell between these two values. Table 2 reports these percentages.

**Table 1:** Descriptive statistics of demographics and outcome variables

Variable	Unweighted N (%)	Weighted N (%)
Age		
12 to 14 years old	5,956 (46.6)	11,894,747 (49.6)
15 to 17 years old	6,823 (53.4)	12,098,402 (50.4)
Gender		
Male	6,611 (51.9)	12,205,206 (51.0)
Female	6,121 (48.1)	11,705,062 (49.0)
Race		
White	8,171 (67.9)	15,781,369 (69.0)
Black	1,892 (15.7)	3,507,535 (15.3)
Other	2,978 (16.4)	3,598,149 (15.7)
Ethnicity		
Hispanic	3,738 (30.4)	5,603,573 (24.3)
Non-Hispanic	8,554 (69.6)	17,500,517 (75.7)
Grade level		
<=7^th^ grade	2,222 (17.4)	4,888,887 (20.4)
8^th^ grade	1,999 (15.7)	3,780,069 (15.8)
9^th^ grade	2,239 (17.6)	3,943,306 (16.5)
10^th^ grade	2,138 (16.8)	3,770,226 (15.8)
11^th^ grade	2,043 (16.0)	3,689,676 (15.4)
Other	2,104 (16.5)	3,852,770 (16.1)
Parent/guardian marital status		
Married	8,036 (63.7)	15,380,103 (65.0)
Widowed, divorced or separated	2,586 (20.5)	4,832,968 (20.4)
Never married	1,990 (15.8)	3,457,895 (14.6)
Annual household income		
<$10,000	860 (7.1)	1,394,024 (6.1)
$10,000 to $24,999	1,858 (15.3)	3,092,480 (13.5)
$25,000 to $49,999	2,777 (22.8)	4,794,317 (21.0)
$50,000 to $99,999	3,154 (25.9)	6,118,112 (26.8)
>=$100,000	3,529 (29.0)	7,425,416 (32.5)

**Table 2:** Percentage of adolescents viewing each type of e-cigarette advertisements

Advertisement Type	Unweighted N (%)	Weighted N (%)
Newspaper	1,474 (11.7)	2,775,899 (11.7)
Radio	980 (7.8)	1,783,605 (7.5)
Billboard	2,110 (16.7)	3,907,131 (16.5)
Television	2,296 (18.2)	4,288,711 (18.1)
Social Media	2,688 (21.3)	5,051,394 (21.3)

Table 3 displays the item response distribution of harm perception. The three harm perception items demonstrated sampling adequacy and reliable estimates for the harm perception factor (Kaiser-Meyer-Olkin Measure of Sampling Adequacy = 0.650, Bartlett’s test of sphericity value *p*<0.05). The factor loadings of the harm perception items ranged from 0.537 to 0.779 with a Cronbach alpha of 0.768. These findings substantiate the calculation of a harm perception factor score. Appendix A displays all three harm perception items’ responses by advertisement types.

**Table 3:** Factor loadings of harm perception items

Variable	Mean	Median	Std Dev	Min	Max
E-cigarette harm perception factor	0.00	0.387	1.000	-3.04	1.36
(1) Harmfulness of nicotine	4.01	4.00	1.049	1	5
(2) Overall harmfulness of e-cigarette	3.40	4.00	0.774	1	4
(3) Harm in ENDS vs. CIGS	1.90	2.00	0.613	1	3

Unadjusted past 30-day exposure to newspaper, radio, billboard, and social media advertising correlated with reduced harm perception, but only the associations for newspaper (*B* = -0.035; 95% CI = -0.164 to -0.055, *p*<0.05) and social media (*B* = -0.088; 95% CI = -0.257 to -0.172, *p*<0.05) were statistically significant. Unadjusted, past 30-day exposure to television advertisements yielded a non-significant association with increased harm perception (*B* = 0.005; 95% CI = -0.033 to 0.058, *p*=0.588). After adjusting for the number of friends who use e-cigarettes, views of people close to you about e-cigarettes, how often you’ve noticed health warnings on e-cigarette packs, and how often you’ve seen anti-tobacco marketing in the past 12 months, the associations between harm perception score and viewing either newspaper (*B* = 0.007; 95% CI = -0.028 to 0.074, *p*=0.378) or social media (*B* = -0.016; 95% CI = -0.080 to 0.002, *p*=0.064) marketing were no longer statistically significant. In contrast, this adjustment revealed a statistically significant association between increased e-cigarette harm perception and exposure to radio (*B* = 0.028; 95% CI = 0.044 to 0.166; *p*<0.05), billboard (*B* = 0.028; 95% CI = 0.031 to 0.119; *p*<0.05), and television (*B* = 0.041; 95% CI = 0.063 to 0.148; *p*<0.05) advertisement. Overall, the covariates reduced association of marketing and harm perception with all forms of media. Tables 4 and 5 provide further detail.

**Table 4:** Linear regression predicting e-cigarette harm perceptions from viewing various types e-cigarette advertisement (without adjustment for covariates)

Advertisement Type	B [95% CI]	*p*-value
Newspaper	-0.035 [-0.162, -0.053]	<0.001
Billboard	-0.014 [-0.084, 0.010]	0.123
Radio	-0.012 [-0.112, 0.019]	0.163
Television	0.005 [-0.033, 0.058]	0.588
Social media	-0.088 [-0.257, -0.172]	<0.001

**Table 5:** Linear regression predicting e-cigarette harm perceptions from viewing various types e-cigarette advertisement (adjusting for these covariates: number of friends using, views of people close to you, frequency of viewing health warning labels in past 30 days, and past 12 month frequency of viewing anti-tobacco advertising)

Advertisement Type	B [95% CI]	p-value
**Newspaper**	**0.007 [-0.028, 0.074]**	**0.378**
Number of friends using ENDS	-0.008 [-0.076, 0.028]	0.370
Views of people close to you	-0.019 [-0.110, -0.006]	0.028
Past 30-day health warning labels noticed	-0.033 [-0.157, -0.048]	<0.001
Past 12 month viewing anti-tobacco ads	-0.018 [-0.111, -0.001]	0.045
**Billboard**	**0.028 [0.031, 0.119]**	**<0.001**
Number of friends using ENDS	0.011 [-0.015, 0.074]	0.193
Views of people close to you	0.000 [-0.046, 0.044]	0.974
Past 30-day health warning labels noticed	-0.012 [-0.080, 0.014]	0.174
Past 12 month viewing anti-tobacco ads	0.005 [-0.034, 0.061]	0.577
**Radio**	**0.028 [0.044, 0.166]**	**<0.001**
Number of friends using ENDS	0.012 [-0.016, 0.108]	0.146
Views of people close to you	0.008 [-0.034, 0.092]	0.365
Past 30-day health warning labels noticed	-0.013 [-0.114, 0.018]	0.152
Past 12 month viewing anti-tobacco ads	-0.004 [-0.082, 0.049]	0.620
**Television**	**0.041 [0.063, 0.148]**	**<0.001**
Number of friends using ENDS	0.018 [0.003, 0.089]	0.003
Views of people close to you	0.024 [0.020, 0.107’	0.004
Past 30-day health warning labels noticed	0.008 [-0.025, 0.066]	0.383
Past 12 month viewing anti-tobacco ads	0.019 [0.005, 0.096]	0.030
**Social media**	**-0.016 [-0.080, 0.002]**	**0.064**
Number of friends using ENDS	-0.038 [-0.134, -0.052]	<0.001
Views of people close to you	-0.063 [-0.195, -0.114]	<0.001
Past 30-day health warning labels noticed	-0.086 [-0.252, -0.166]	<0.001
Past 12 month viewing anti-tobacco ads	-0.068 [-0.208, -0.122]	<0.001

## Discussion

Low harm perceptions of non-cigarette tobacco products predict new use of these products [19]. Previous research about e-cigarette marketing overwhelmingly indicates that adolescents are more likely to perceive e-cigarettes as less harmful following exposure to advertisements across all types. However, the majority of these studies fail to adjust for the possible impact of exposure and environmental variables. Before adjusting for these covariates, our findings generally aligned with previous research and found a large and significant association between exposure to social media and newspaper advertisements and a reduction in adolescent perception of e-cigarette harms. For radio, billboard, and television advertisements, there was also an association between exposure and lowered harm perception among adolescents but this did not achieve statistical significance. Taken together, these findings suggest that various types of advertisement exposure can lower harm perception of e-cigarettes among adolescents.

After adjusting for covariates such as exposure to anti-tobacco messaging and the number of friends using e-cigarettes, social media and newspaper advertising continued to exhibit a strong association with lowered perception of harm by adolescents.. This suggests that social media and newspaper marketing are an effective way to influence adolescents and makes recent research identifying influencer e-cigarette marketing as a popular social media tactic troubling [20, 21]. Through this method, individuals with large social media followings include the product in their posts, even if a formal sponsorship is not explicitly mentioned. Although the Federal Trade Commission (FTC) does not view social media advertising as substantively different from traditional advertising from a regulatory perspective [22], regulating e-cigarette promotions through social media may prove challenging. Of particular concern are promotional messages integrated into and presented as non-commercial content on social media. (Commission) Our results support the value of the FTC asking e-cigarette manufacturers to submit information about their marketing practices [23]. As for newspapers, similar subtle marketing tactics are used. Images of attractive people using e-cigarettes are included, appealing to an adolescent’s desire to fit in by emulating individuals they perceive as popular or inspirational. To better understand the underlying mechanisms of adolescent susceptibility to e-cigarette advertising, further research, particularly on the mechanisms of influencer marketing, is essential.

A second key finding was that the number of friends using e-cigarettes, family attitude toward product use, exposure to warning labels, and exposure to anti-tobacco advertisements all significantly reduced the negative correlation between the predictor and outcome variables for all advertising media. Though product advertising influences harm perception, our findings suggest that peer/family influence and exposure to dissenting marketing mitigates the impact of e-cigarette marketing and highlights the importance of anti-tobacco advertisements and warning labels. A more family-centered approach where policy makers aim to not only educate adolescents but also influence their friends and families may also be beneficial.

While we identified links to factors that mitigate harm perceptions, future research is needed to determine which factors are most influential, and what forms of regulation are most effective in curbing use. For example, there is benefit in assessing prevention strategies by the United States Food and Drug Administration’s (US FDA) Center for Tobacco Products (CTP) [24]. Responding to the marked increase in adolescent e-cigarette use, the FDA and several state governments implemented anti-vaping policies. In 2019, the FDA banned the sale of all e-cigarette flavors except for tobacco and menthol [25]. Furthermore, several states increased e-cigarette taxes to discourage e-cigarette purchase, and in recent years, the FDA has issued warning letters about misleading advertising and labeling [26]. Others advocate the use of messaging that questions the safety of e-cigarettes, but a recent study found that this fails to curb e-cigarette use [27]. Further research about the impact of these regulations and other factors can facilitate developing more targeted and effective approaches to curtailing e-cigarette appeal.

### Limitations

This study has several limitations. First, the PATH Study data were self-reported and potentially subject to bias. Respondents might answer with what they believe is most acceptable rather than the truth. Second, in assessing their exposure to each form of advertisement, respondents were asked to answer “Yes” or “No” rather than indicate the frequency in which they viewed these advertisements. This prevents any differentiation between respondents who viewed a type of advertisement once versus multiple times. There may be a dose-response curve to the impact of advertising that this study failed to capture. Additionally, we were unable to ascertain whether certain types of advertisements were observed in higher amounts than others.

### Conclusion

This study re-affirmed that e-cigarette marketing influences adolescent harm perception, particularly social media and newspaper advertising. However, adjusting for covariates pertaining to environmental support and exposure to anti-tobacco marketing weakened this association across all marketing forms. This suggests that increasing anti-tobacco marketing and incorporating initiatives with holistic, family-centered educational approaches could potentially curb e-cigarette use among adolescents.

## Data Availability

The data are available to the public via the PATH website: https://www.drugabuse.gov/research/nida-research-programs-activities/population-assessment-tobacco-health-path-study.

## Appendix

Table 6

**Table 6:** Descriptive statistics of the e-cigarette harm perception items across different advertisement types

Variable	Unweighted N (%)	Weighted N (%)
**Newspaper**		
Harmfulness of nicotine		
Not at all	27 (1.8)	56,650 (2.0)
Slightly	114 (7.6)	218,572 (7.8)
Somewhat	345 (23.2)	636,325 (22.7)
Very	437 (29.4)	833,492 (29.8)
Extremely	564 (37.9)	1,054,044 (37.7)
Overall harmfulness of e-cigarette		
No harm	33 (2.2)	61,574 (2.2)
Little harm	197 (13.3)	388,634 (13.9)
Some harm	494 (33.3)	923,160 (33.0)
A lot of harm	761 (51.2)	1,424,404 (50.9)
Harm in ENDS vs. CIGS		
Less harmful	435 (29.4)	819,362 (29.4)
About the same	855 (57.8)	1,615,997 (58.0)
A lot of harm	190 (12.8)	350,171 (12.6)
**Billboard**		
Harmfulness of nicotine		
Not at all	33 (1.6)	55,358 (1.4)
Slightly	131 (6.2)	254,318 (6.5)
Somewhat	492 (23.1)	887,106 (22.5)
Very	609 (28.6)	1,149,091 (29.2)
Extremely	861 (40.5)	1,591,467 (40.4)
Overall harmfulness of e-cigarette		
No harm	40 (1.9)	67,433 (1.7)
Little harm	232 (10.9)	434,828 (11.1)
Some harm	716 (33.7)	1,319,481 (33.5)
A lot of harm	1136 (53.5)	2,112,497 (53.7)
Harm in ENDS vs. CIGS		
Less harmful	566 (26.7)	1,084,318 (27.6)
About the same	1306 (61.6)	2,391,732 (60.9)
A lot of harm	248 (11.7)	454,304 (11.6)
**Radio**		
Harmfulness of nicotine		
Not at all	31 (3.2)	46,361 (2.6)
Slightly	71 (7.2)	140,359 (7.8)
Somewhat	240 (24.4)	429,542 (24.0)
Very	261 (26.5)	479,788 (26.8)
Extremely	381 (38.7)	693,613 (38.8)
Overall harmfulness of e-cigarette		
No harm	31 (3.1)	57,132 (3.2)
Little harm	127 (12.9)	233,820 (13.0)
Some harm	305 (30.9)	557,413 (31.0)
A lot of harm	525 (53.1)	951,541 (52.9)
Harm in ENDS vs. CIGS		
Less harmful	218 (22.1)	409,626 (22.8)
About the same	614 (62.3)	1,119,477 (62.4)
A lot of harm	154 (15.6)	266,187 (14.8)
**Television**		
Harmfulness of nicotine		
Not at all	43 (1.9)	72,040 (1.7)
Slightly	144 (6.2)	277,395 (6.4)
Somewhat	537 (23.2)	1,002,990 (23.2)
Very	657 (28.4)	1,216,562 (28.2)
Extremely	931 (40.3)	1,749,651 (40.5)
Overall harmfulness of e-cigarette		
No harm	53 (2.3)	101,649 (2.3)
Little harm	269 (11.6)	504,151 (11.6)
Some harm	747 (32.2)	1,369,363 (31.6)
A lot of harm	1249 (53.9)	2,356,451 (54.4)
Harm in ENDS vs. CIGS		
Less harmful	501 (21.7)	980,522 (22.7)
About the same	1462 (63.2)	2,717,059 (62.9)
A lot of harm	349 (15.1)	624,491 (14.4)
**Social Media**		
Harmfulness of nicotine		
Not at all	55 (2.0)	106,670 (2.1)
Slightly	219 (8.1)	425,580 (8.4)
Somewhat	682 (25.2)	1,262,363 (24.8)
Very	777 (28.7)	1,484,858 (29.2)
Extremely	971 (35.9)	1,805,626 (35.5)
Overall harmfulness of e-cigarette		
No harm	58 (2.1)	105,854 (2.1)
Little harm	375 (13.9)	731,855 (14.4)
Some harm	996 (36.8)	1,872,869 (36.8)
A lot of harm	1277 (47.2)	2,375,434 (46.7)
Harm in ENDS vs. CIGS		
Less harmful		
About the same		
A lot of harm		

## Abbreviations

CTP: Center for Tobacco Products
FTC: Federal Trade Commission
PATH: Population Assessment of Tobacco and Health
US FDA: United States Food and Drug Administration
